# Real-world management of non-alcoholic steatohepatitis differs from clinical practice guideline recommendations and across regions

**DOI:** 10.1016/j.jhepr.2021.100411

**Published:** 2021-11-22

**Authors:** Quentin M. Anstee, Kate Hallsworth, Niall Lynch, Adrien Hauvespre, Eid Mansour, Sam Kozma, John-Paul Marino, Juliana Bottomley, James Piercy, Victoria Higgins

**Affiliations:** 1 Translational & Clinical Research Institute, Faculty of Medical Sciences, Newcastle University, Newcastle upon Tyne, UK; 2 Newcastle National Institute for Health Research Biomedical Research Centre, Newcastle upon Tyne Hospitals NHS Foundation Trust, Newcastle upon Tyne, UK; 3 Gilead Sciences, Hayes, Uxbridge, UK; 4 Gilead Sciences Middle East, Dubai, United Arab Emirates; 5 Gilead Sciences, Mississauga, Canada; 6 Adelphi Real World, Bollington, Cheshire, UK

**Keywords:** Non-alcoholic steatohepatitis, liver disease, clinical practice guidelines, diagnostic pathways, patient management, AASLD, American Association for the Study of Liver Diseases, ALT, alanine aminotransferase, AST, aspartate aminotransferase, EASD, European Association for the Study of Diabetes, EASL, European Association for the Study of the Liver, EASO, European Association for the Study of Obesity, EU5, France, Germany, Italy, Spain and United Kingdom, FIB-4, Fibrosis-4, HbA_1c_, glycated hemoglobin, NAFLD, non-alcoholic fatty liver disease, NASH, non-alcoholic steatohepatitis, NIT, non-invasive test, T2DM, type 2 diabetes mellitus, VCTE, vibration-controlled transient elastography

## Abstract

**Background & Aims:**

Despite availability of diagnostic and management reference guidelines outlining standard of care for patients with non-alcoholic fatty liver disease (NAFLD) and non-alcoholic steatohepatitis (NASH), national and regional guidelines are lacking, resulting in variations in patient management between regions. We retrospectively analyzed patient characteristics and management data from the Adelphi Real World NASH Disease Specific Programme™ for patients with NASH in the EU5, Canada, and the Middle East to identify gaps between real-world practice and that advocated by reference guidelines, irrespective of clinician awareness or consultation of guidelines.

**Methods:**

We performed an analysis of physicians (hepatologists, gastroenterologists, diabetologists) and their patients diagnosed with NASH. Physicians completed patient record forms for the next 5 consulting patients, collecting information on patient care, including diagnosis and disease management.

**Results:**

A total of 429 physicians provided data for 2,267 patients with NASH (EU5, n = 1,844; Canada, n = 130; Middle East, n = 293). Patient age, physician-defined fibrosis stage, comorbidities and symptoms, and diagnostic testing practices highlighted statistically significant differences across regions. Substantial disconnects between reference guidelines and real-world practice were observed. Use of liver function tests, non-invasive tests (*e.g.* ultrasound and transient elastography), and tests to exclude other conditions was suboptimal. Although lifestyle advice was widely provided, patients were less commonly referred to diet, exercise, and lifestyle specialists. Two-thirds of patients were receiving off-label treatment for NASH or associated underlying conditions with the aim of improving NASH, most commonly statins, metformin, and vitamin E.

**Conclusion:**

Real-world NASH management approaches differ across regions and from proposed standard of care represented by reference multidisciplinary guidelines. Establishment and awareness of, and adherence to regional and national guidelines may improve identification and management of patients with NASH and potentially improve outcomes in this population.

**Lay summary:**

Although reference guidelines are available to guide the management of patients with NASH, these are not widely used and there is a lack of national guidelines. Our study shows how clinical practice in the EU, Canada, and Middle East differs from proposed standard of care, particularly relating to how patients are diagnosed and treated. Wider establishment of, awareness of, and reference to guidelines may improve how physicians identify and manage patients with NASH.

## Highlights

• Reference guidelines exist for the diagnosis and management of patients with NASH.
• This analysis compared reference guidelines and real-world practice in 3 regions.
• Substantial deviations from reference guidelines were seen in testing and treatment.
• Referral to diet, exercise, and lifestyle specialists was suboptimal.
• Establishment, awareness, and adherence to national guidelines is needed.

## Graphical abstract

**Figure:**
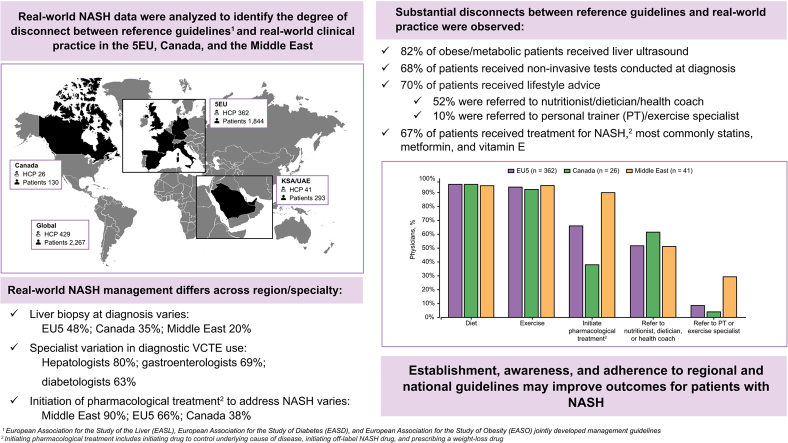

## Introduction

Non-alcoholic steatohepatitis (NASH) is the progressive form of non-alcoholic fatty liver disease (NAFLD), an increasingly common disease associated with metabolic syndrome, which can lead to cirrhosis, hepatocellular carcinoma, and substantial morbidity.[1], [2], [3] NAFLD/NASH carries a substantial socioeconomic burden which, coupled with rising prevalence, is an underrecognized but growing public health challenge.^4^^,^^5^ Careful management of patients with NASH is important, as reversal of NASH-associated fibrosis is possible but requires patients to make significant lifestyle changes targeting weight loss through dietary modification and increased physical activity.^6^^,^^7^ Sustaining changes to maintain weight loss is difficult.^8^ In their recent review, Povsic *et al.* found no reports of sustained weight loss and impact on outcomes in people with NASH.^9^ Pharmacological intervention may also be needed,^10^ although the success of such approaches depends on the extent of liver damage and expected treatment outcomes. There are currently no Food and Drug Administration/European Medicines Agency-approved NASH-specific pharmacological therapies, although substantial effort is ongoing to develop treatments, with many agents in clinical development.

Diagnosis of NASH can be challenging.^11^ Liver biopsy is currently the reference standard for identifying steatohepatitis and subsequently grading and staging disease, although its use is limited by cost, patient refusal, and specialist pathologist interpretation.^12^ In the real-world setting, physicians may not have access to or wish to use this invasive diagnostic method. Alternative non-invasive tests (NITs) have been developed for the diagnosis of liver fibrosis. These generally have high negative predictive value and can effectively rule out advanced fibrosis. NITs include a range of biomarker and physical techniques.^13^ Composite scores such as the NAFLD fibrosis score and Fibrosis-4 (FIB-4) index provide cost-effective, non-invasive, and efficient risk stratification to identify patients warranting specialist referral or further testing.^14^ In particular, the FIB-4 index has shown promising results.^15^^,^^16^ Vibration-controlled transient elastography (VCTE) is subject to some limitations, including potentially reduced accuracy of staging in patients who are older, or exhibit obesity, hypertension, or type 2 diabetes mellitus (T2DM), but it complements the simple panel tests.^17^

Given the increasing prevalence of NASH, the European Association for the Study of the Liver (EASL), European Association for the Study of Diabetes (EASD), and European Association for the Study of Obesity (EASO) jointly developed management guidelines.^18^ Levels of real-world uptake and usage of these guidelines have not been established and clinical practice guidelines are not available in many countries and regions, including some European countries, Canada, and the Middle East.^5^^,^^19^^,^^20^ We undertook an analysis across the EU5 (France, Germany, Italy, Spain, and United Kingdom), Canada, and the Middle East (United Arab Emirates and Kingdom of Saudi Arabia) to assess how management practices aligned with a standard of care recommended by international reference guidelines, irrespective of region.^18^ Although these guidelines may not be adopted in full in non-European territories, the majority of the recommendations assessed are well aligned to those in the American Association for the Study of Liver Diseases (AASLD) guidelines^12^ and represent a reasonable standard of care that should be aimed for as a benchmark, regardless of the region or healthcare system. A secondary objective was to identify any differences between management approaches across regions.

## Patients and methods

### Study design and participants

Data were drawn from the 2018 Adelphi Real World NASH Disease Specific Programme^TM^ conducted in EU5, Canada, and Middle East. The Disease Specific Programme is a point-in-time survey of physicians and their patients presenting in real-world clinical settings, as described previously.[21], [22], [23]

Secondary care physicians (hepatologists, gastroenterologists, or diabetologists) managing ≥10 patients/month with NASH and personally responsible for NASH clinical management decisions were eligible. Patients were ≥18 years old, with physician-confirmed diagnosis of NASH (liver biopsy or NIT). Patients were not participating in clinical trials at data collection.

The study was performed in accordance with relevant guidelines; ethics approval was obtained from Freiburg Ethics Commission International (Approval No. 017/1931) for EU5 and Middle East, and the Western International Review Board (Tracking No. 20180044) for Canada. Patients provided written informed consent for use of their anonymized and aggregated data.

### Sample and data collection

Physicians completed an online survey capturing views on patient management, and questionnaires for the next 5 consecutive patients with NASH (10 in the Middle East) presenting for routine care. The questionnaire collected patient demographic and clinical information including patient care, diagnosis, metabolic conditions (defined as T2DM, metabolic syndrome, dyslipidemia, hypertension, insulin resistance, or any combination of the above), and disease management.

All authors had access to survey data and reviewed and approved the final manuscript.

### Statistical analyses

Analyses were performed in 2 stages. The first involved comparison of patients across the EU5, Canada, and the Middle East for demographics, clinical characteristics, tests, and treatment, particularly with reference to selected guideline recommendations. Numeric variables were compared using frequency, mean, standard deviation (SD), and/or range within each region, and performing analysis of variance test (*p* value reported) across regions. Categorical variables were compared using the number of patients within each category and the proportion of total (%) by region and performing a χ^2^ test (*p* value reported) across regions. *p* <0.05 was considered statistically significant.

The second stage analyzed regions separately by comparing the aforementioned variables between 2 subgroups (within each region):

• Liver biopsy (patients with *vs.* without);
• Physician-stated fibrosis stage (F0–F2 *vs.* F3/F4).

Numeric variables were compared using frequency, mean, SD, and/or range within each subgroup, and performing a *t* test or analysis of variance test (*p* value reported) across subgroups. Categorical variables were compared by providing the frequency within each category and proportion of total by subgroup and performing Fisher’s exact test for 2×2 cases or χ^2^ test (*p* value reported) across subgroups.

## Results

### Physicians and patients

**Fig. 1:**
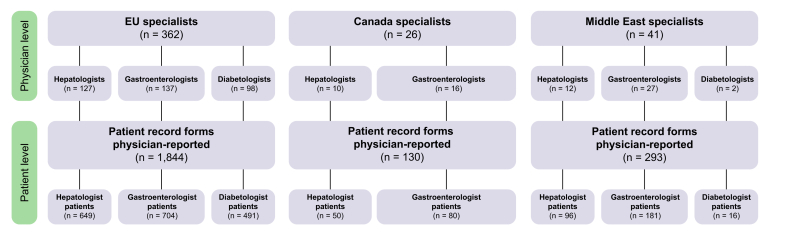
Patients and physicians. Healthcare practitioner breakdowns reflect national healthcare systems. The hepatologist sample includes hepatologists in France, Italy, UK, and the Middle East, gastroenterologists with a sub-specialty in hepatology in Germany, Spain, and Canada, and hepato-gastroenterologists in France. The Canadian sample did not include diabetologists/endocrinologists.

Overall, 429 physicians (hepatologists, n = 149 [35%], gastroenterologists, n = 180 [42%], and diabetologists, n = 100 [23%]) provided data on 2,267 patients with NASH (EU5, n = 1,844 [81%]; Canada, n = 130 [6%]; Middle East, n = 293 [13%]; Fig. 1; Supplementary results). Patient characteristics are shown in Table 1. Statistically significant regional differences were observed, including age, prevalence of physician-stated cirrhosis (F4 fibrosis stage), symptoms at diagnosis, and disease status. Not all patients were assigned a fibrosis score. Where fibrosis stage was recorded, most patients were currently classified with early fibrosis (F0–2; 77%).

**Table 1:** **Patient demographics and clinical characteristics**.

Characteristic	EU5 (n = 1,844)	Canada (n = 130)	Middle East (n = 293)	*p* value
Median age, years (range)	56 (18–89)	55 (19–87)	45 (20–87)	<0.0001^d^
Sex, n (%)				0.5526^e^
Male	1,137 (62)	74 (57)	178 (61)	
Female	707 (38)	56 (43)	115 (39)	
Ethnicity				<0.0001^e^
White/Caucasian	1,612 (87)	106 (82)	0	
Middle Eastern	21 (1)	2 (2)	288 (98)	
Asian Indian	68 (4)	7 (5)	5 (2)	
Hispanic Latino	52 (3)	2 (2)	0	
Afro-Caribbean	31 (2)	0	0	
North African	29 (2)	0	0	
Other	31 (2)	13 (10)	0	
Mean body mass index, kg/m^2^ (SD)	32.6 (6.5)	32.2 (6.6)	31.9 (15.0)	0.3811^d^
Mean time since diagnosis, years (SD)	1.5 (2.3)	1.5 (2.1)	1.8 (1.7)	0.0510^d^
Diagnosing HCP, n (%)				<0.0001^e^
Gastroenterologist	701 (38)	75 (58)	190 (65)	
Hepatologist	727 (39)	45 (35)	91 (31)	
Diabetologist/endocrinologist	317 (17)	0	3 (1)	
GP/PCP	43 (2)	9 (7)	1 (<1)	
Other healthcare practitioner^a^	20 (1)	1 (1)	7 (2)	
Do not know/missing	36 (2)	0	1 (<1)	
Mean no. of NASH consultations (SD)	4.3 (4.3)	3.4 (2.4)	3.6 (1.4)	0.0013^d^
Lean NASH, n (%)^b^	131 (7)	11 (8)	34 (12)	0.0267^e^
Physician-stated current fibrosis stage, n (%)				<0.001^e^
F0	204 (11)	18 (14)	22 (8)	
F1	565 (31)	18 (14)	117 (40)	
F2	378 (20)	11 (8)	56 (19)	
F3	203 (11)	10 (8)	45 (15)	
F4	118 (6)	25 (19)	23 (8)	
Missing	376 (20)	48 (37)	30 (10)	
NASH status, n (%)				<0.0001^e^
Improving	278 (15)	25 (19)	120 (41)	
Stable	1,128 (61)	75 (58)	118 (40)	
Deteriorating slowly	362 (20)	24 (18)	48 (16)	
Deteriorating rapidly	40 (2)	4 (3)	2 (1)	
Fluctuating	36 (2)	2 (2)	5 (2)	
Comorbidities, n (%)^c^				
T2DM	1,083 (59)	51 (39)	129 (44)	<0.0001^e^
Hypertension	892 (48)	43 (33)	104 (35)	<0.0001^e^
Dyslipidemia	847 (46)	44 (34)	119 (41)	0.0096^e^
Metabolic syndrome	455 (25)	21 (16)	68 (23)	0.084^e^
Insulin resistance	291 (16)	18 (14)	37 (13)	0.3401^e^
Any of the above	1,477 (80)	90 (69)	230 (78)	0.0120^e^
Symptoms at diagnosis, n (%)^c^	*(n = 1,425)*	*(n = 91)*	*(n = 267)*	
Fatigue	886 (62)	63 (69)	219 (82)	<0.0001^e^
Sleep disturbance	532 (37)	30 (33)	23 (9)	<0.0001^e^
General weakness	529 (37)	33 (36)	190 (71)	<0.0001^e^
Swelling of stomach/abdomen	341 (24)	7 (8)	19 (7)	<0.0001^e^
Swelling in legs, ankles, feet	335 (24)	10 (11)	30 (11)	<0.0001^e^
Itchy skin	212 (15)	1 (1)	3 (1)	<0.0001^e^
Insomnia	194 (14)	11 (12)	9 (3)	<0.0001^e^
Confusion/difficulty concentrating	147 (10)	4 (4)	22 (8)	0.1231^e^

GP, general practitioner; HCP, healthcare practitioner; NASH, non-alcoholic steatohepatitis; PCP, primary care physician; T2DM, type 2 diabetes mellitus.

a Includes infectious disease or exercise specialist, specialist nurse, radiologist, cardiologist, and dietician.

b Physician-reported.

c Most common current symptoms/comorbidities.

d Analysis of variance test.

e χ^2^ test.

Patient symptoms according to physician-reported fibrosis score are detailed in Table S1. In Canada and the Middle East, swelling in legs, ankles, and feet, and fatigue were statistically significantly more common in patients with advanced *vs.* early fibrosis.

### Alignment of real-world practice with diagnostic and treatment guidelines

The cross-specialty EASL-EASD-EASO guidelines specify a number of recommended actions.^18^ Seven of these address diagnostic work-up, comorbidity assessment, and therapy and were selected as yardsticks against which our real-world data were compared.

(1) *Guideline: In subjects with obesity or metabolic syndrome, screening for NAFLD by liver enzymes and/or ultrasound should be part of routine work-up. Ultrasound is the preferred first-line diagnostic procedure for imaging of NAFLD, as it provides additional diagnostic information*

Standard clinical chemistry assessments (aspartate aminotransferase [AST], alanine aminotransferase [ALT], alkaline phosphatase, and bilirubin) were performed in 80% (n = 1,433), 85% (n = 1,526), 60% (n = 1,073), and 58% (n = 1,045) of 1,797 patients with metabolic conditions, respectively. Testing rates were lower in the EU5 than in Canada or the Middle East. Liver ultrasound, which should be part of standard work-up, was performed in 82% of patients with metabolic conditions, although 99% of patients in the Middle East underwent this test.

*(2) Guideline: Individuals with steatosis should be screened for secondary causes of NAFLD, including a careful assessment of alcohol intake. Other chronic liver diseases that may coexist with NAFLD should be identified as this might result in more severe liver injury.*

Overall, 1,658 of 2,267 patients (73%) underwent serological tests for viral hepatitis; 54 (2%) had positive hepatitis B or C results. ‘Screening’ tests to eliminate less common causes of steatosis were performed infrequently (Table 2). Serum ferritin and serum ceruloplasmin were most often used in Canada; assessment of associated conditions, *e.g.* T2DM, obesity, and impaired glucose tolerance, was most likely in the Middle East (Table S2). Alcohol consumption was assessed in 1,933 of 1,974 patients (98%) in the EU5 and Canada but not in the Middle East for religious reasons.

**Table 2:** **Diagnostic and monitoring tests**.

Test	EU5 (n = 1,844)	Canada (n = 130)	Middle East (n = 293)	*p* value
Mean no. of diagnostic and exclusion tests (SD)^a^	19.8 (9.2)	23.4 (7.6)	22.1 (6.9)	<0.0001^c^
Diagnostic tests, n (%)				
AST	1,349 (73)	109 (84)	268 (91)	<0.0001^d^
ALT	1,455 (79)	124 (95)	268 (91)	<0.0001^d^
Alkaline phosphatase	956 (52)	111 (85)	257 (88)	<0.0001^d^
Bilirubin	953 (52)	109 (84)	226 (77)	<0.0001^d^
HbA_1c_	1,292 (70)	98 (75)	185 (63)	<0.0001^d^
Total cholesterol	1,398 (76)	91 (70)	267 (91)	<0.0001^d^
HDL-cholesterol	1,371 (74)	91 (70)	270 (92)	<0.0001^d^
Liver biopsy	876 (48)	45 (35)	60 (20)	<0.0001^d^
VCTE	1,101 (60)	57 (44)	211 (72)	<0.0001^d^
FIB-4 index	105 (6)	6 (5)	7 (2)	0.0580^d^
Liver ultrasound	1,462 (79)	101 (78)	280 (96)	<0.0001^d^
Tests of exclusion				
Serological tests for absence of viral hepatitis	1,308 (71)	107 (82)	243 (83)	<0.0001^d^
Gilbert’s syndrome	463 (25)	48 (37)	29 (10)	<0.0001^d^
Paget’s disease	301 (16)	19 (15)	20 (7)	<0.0001^d^
Wilson’s disease	555 (30)	65 (50)	49 (17)	<0.0001^d^
Celiac disease	557 (30)	59 (45)	35 (12)	<0.0001^d^
NIT assessing disease severity^b^	1,210 (66)	71 (55)	255 (87)	<0.001^d^
Monitoring tests				
Mean no. of monitoring tests (SD)	19.8 (9.2)	23.4 (7.6)	22.1 (6.9)	<0.0001^c^

ALT, alanine aminotransferase; AST, aspartate aminotransferase; BARD, BMI, AST:ALT ratio, and diabetes; EU5, France, Germany, Italy, Spain, and UK; FIB-4, Fibrosis-4; HbA_1c_, glycated hemoglobin; HDL, high-density lipoprotein; NAFLD, non-alcoholic fatty liver disease; NIT, non-invasive test; VCTE, vibration-controlled transient elastography.

a Includes all diagnostic and elimination tests.

b Includes VCTE, FibroTest, serum enhanced liver fibrosis test, NAFLD Fibrosis Score, FIB-4 Index, and BARD Score.

c Analysis of variance test.

d Chi-square test.

Thirteen patients had cancer diagnoses (tumor type not specified), suggesting that hepatocellular cancer was rare in this sample.

*(3) Guideline: All individuals with steatosis should be screened for features of metabolic syndrome (i.e. T2DM, dyslipidemia, hypertension, and obesity), independent of liver enzymes. In persons with NAFLD, screening for diabetes is mandatory, by fasting or random blood glucose or HbA_1c_*

Performance of these and other screening tests varied significantly across regions (Table 2). Total cholesterol was measured in 1,756 patients (77%), 1,070 (65%) of whom were receiving statins; glycated hemoglobin (HbA_1c_) was measured in 1,575 patients (69%). Only 219 of 338 patients (65%) considered high risk for cardiovascular events based on prior myocardial infarction, congestive heart failure, peripheral vascular disease, or cerebrovascular disease were receiving statins.

*(4) Guideline: Biomarkers and scores of fibrosis, as well as transient elastography, are acceptable non-invasive procedures for the identification of cases at low risk of advanced fibrosis/cirrhosis*

NITs gauging disease severity were conducted at diagnosis in 1,536 patients (68%), with regional differences (Table 2). Only 118 patients (5%) had physician-reported FIB-4 results, despite the elements required for FIB-4 frequently being collected and this being one of the best-performing and most readily available ‘simple’ scores. When availability of the individual FIB-4 components was assessed, 875 EU5 (47%), 97 Canadian (75%), and 256 Middle Eastern patients (87%) had information available to calculate the FIB-4 index, suggesting that lack of available resource or access to requisite tests was not the cause of FIB-4 underuse. Only 60% of patients underwent VCTE; VCTE use varied statistically significantly across regions (Table 2). VCTE and liver biopsy use also varied according to physician specialty: 80%, 69%, and 63% of hepatologists, gastroenterologists, and diabetologists, respectively, reported using VCTE to diagnose their patients with NASH; 66%, 61%, and 57% of hepatologists, gastroenterologists, and diabetologists, respectively, used liver biopsy for diagnostic purposes.

*(5) Guideline: NASH has to be diagnosed by a liver biopsy showing steatosis, hepatocyte ballooning, and lobular inflammation*

Liver biopsy was used in a minority of patients. A statistically significant regional difference in diagnostic liver biopsies was observed, these being more widely used in the EU5 *vs*. Canada and the Middle East (48% *vs.* 35% and 20% of patients, respectively; Table 3). Likely reflecting an element of selection before biopsy, liver biopsy was more common in patients with more advanced fibrosis scores in the EU5 and Middle East, although not in Canada (Table S2). Among patients who had NITs to gauge disease severity at diagnosis, 311 of 973 patients (32%) classified as low risk (*i.e*., having early fibrosis) still underwent liver biopsy. Conversely, 157 of 320 patients (49%) classified as at high risk for advanced fibrosis did not undergo liver biopsy.

*(6) Guideline: Structured programs aimed at lifestyle changes towards healthy diet and habitual physical activity are advisable in NAFLD*

**Table 3:** **Patient characteristics according to region and liver biopsy status (either at diagnosis or post-diagnosis)**.

Characteristic	EU5 (n = 1,844)			Canada (n = 130)			Middle East (n = 293)		
	Liver biopsy (n = 893)	No liver biopsy (n = 951)	*p* value	Liver biopsy (n = 46)	No liver biopsy (n = 84)	*p* value	Liver biopsy (n = 65)	No liver biopsy (n = 228)	*p* value
Mean age (SD)	56 (11)	56 (12)	0.7799^a^	53 (13)	57 (14)	0.1294^a^	**52 (16)**	**46 (17)**	**0.0102**^a^
Body mass index >30 kg/m^2^, n (%)	**644 (72)**	**636 (67)**	**0.0152**^b^	28 (61)	48 (57)	0.7132^b^	**47 (72)**	**95 (42)**	**<0.0001**^b^
NASH duration	**1.7 (2.7)**	**1.3 (1.9)**	**0.0010**^a^	1.6 (2.0)	1.4 (2.1)	0.4952^a^	1.9 (1.0)	1.8 (1.9)	0.5378^a^
Metabolic condition									
T2DM	540 (60)	543 (57)	0.1427^b^	22 (48)	29 (35)	0.1883^b^	30 (46)	99 (43)	0.7772^b^
Metabolic syndrome	**246 (28)**	**209 (22)**	**0.0058**^b^	8 (17)	13 (15)	0.8062^b^	**9 (14)**	**59 (26)**	**0.0462**^b^
Dyslipidemia	422 (47)	425 (45)	0.2823^b^	18 (39)	26 (31)	0.4384^b^	29 (45)	90 (39)	0.4766^b^
Hypertension	**484 (54)**	**408 (43)**	**<0.001**^b^	13 (28)	30 (36)	0.4394^b^	21 (32)	83 (36)	0.5611^b^
Insulin resistance	144 (16)	147 (15)	0.7018^b^	6 (13)	12 (14)	1.0000^b^	11 (17)	26 (11)	0.2886^b^
Any of the above	**750 (84)**	**727 (76)**	0.0001^b^	31 (67)	59 (70)	0.8428^b^	54 (83)	176 (77)	0.3925^b^
Diagnosing HCP, n (%)			**0.0056**^c^			**0.0160**^c^			0.0974^b^
Hepatologist	**353 (40)**	**374 (39)**		**23 (50)**	**22 (26)**		28 (43)	63 (28)	
Gastroenterologist	**350 (39)**	**351 (37)**		**19 (41)**	**56 (67)**		36 (55)	154 (68)	
Diabetologist	**159 (18)**	**158 (17)**		**0**	**0**		0	3 (1)	
Other	**17 (2)**	**46 (5)**		**4 (9)**	**6 (7)**		1 (2)	7 (3)	
Missing	**14 (2)**	**22 (2)**		**0**	**0**		0	1 (<1)	
NASH condition status, n (%)			**0.0002**^c^			0.1771^c^			**<0.0001**^c^
Improving	**131 (15)**	**147 (15)**		11 (24)	14 (17)		**28 (43)**	**92 (40)**	
Stable	**510 (57)**	**618 (65)**		29 (63)	46 (55)		**10 (15)**	**108 (47)**	
Deteriorating slowly	**214 (24)**	**148 (16)**		4 (9)	20 (24)		**22 (34)**	**26 (11)**	
Deteriorating rapidly	**22 (2)**	**18 (2)**		2 (4)	2 (2)		**2 (3)**	**0**	
Fluctuating	**16 (2)**	**20 (2)**		0	2 (2)		**3 (5)**	**2 (1)**	
Current VCTE result, mean (SD)	**21 (17)**	**17 (15)**	**<0.0001**^a^	16 (17)	11 (8)	0.1449^a^	**21 (15)**	**14 (10)**	**0.0002**^a^
Physician-reported fibrosis stage at diagnosis	*(n = 711)*	*(n = 700)*	**<0.0001**^c^	*(n = 41)*	*(n = 49)*	**0.0236**^c^	*(n = 62)*	*(n = 210)*	**<0.0001**^c^
F0	**57 (8)**	**121 (17)**		**5 (12)**	**11 (22)**		**0**	**46 (22)**	
F1	**255 (36)**	**238 (34)**		**10 (24)**	**5 (10)**		**20 (32)**	**110 (52)**	
F2	**210 (30)**	**198 (28)**		**10 (24)**	**8 (16)**		**8 (13)**	**38 (18)**	
F3	**136 (19)**	**81 (12)**		**10 (24)**	**6 (12)**		**26 (42)**	**13 (6)**	
F4	**53 (7)**	**62 (9)**		**6 (15)**	**19 (39)**		**8 (13)**	**3 (1)**	

NOTE: Values highlighted in bold indicate statistically significant differences between patients with a liver biopsy *vs*. those without.

HCP, healthcare provider; NASH, non-alcoholic steatohepatitis; T2DM, type 2 diabetes mellitus; VCTE, vibration-controlled transient elastography.

a *t* test.

b Fisher’s exact test.

c χ^2^ test.

**Fig. 2:**
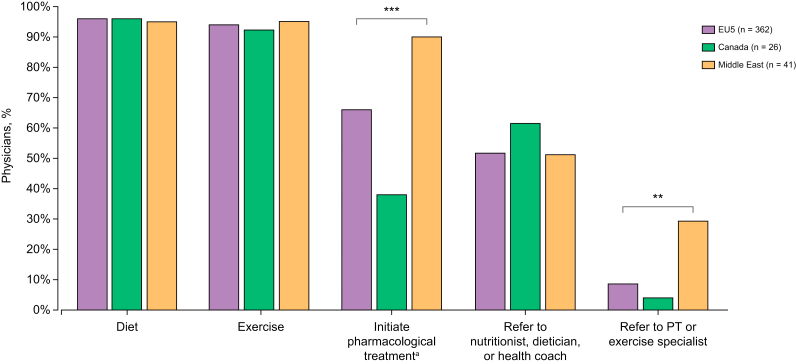
Physician-reported NASH management approaches (% physicians). ∗∗*p*<0.001; ∗∗∗*p*<0.0001 (χ^2^ test). ^a^Initiating pharmacological treatment includes initiating drug to control underlying cause of disease, initiating off-label NASH drug, and prescribing a weight-loss drug. EU5, France, Germany, Italy, Spain, and UK; NASH, non-alcoholic steatohepatitis; PT, personal trainer.

Physicians adopted a variety of approaches in managing patients with NASH (Fig. 2). Targeting weight loss through diet and exercise was advocated by physicians across all regions. Overall, 70% of patients received lifestyle advice, although only 52% were referred to nutritionists/dieticians/health coaches and 10% to exercise specialists, suggesting that almost half had not received adequate support to make necessary lifestyle changes. Referral to nutritionists/dieticians/health coaches was more common in Canada (62% of physicians) than in the EU5 (52%) or Middle East (51%); referral to exercise specialists was most common in the Middle East.

*(7) Guideline: Pharmacotherapy should be reserved for patients with NASH, particularly for those with significant fibrosis (stage F2 and higher). While no firm recommendations can be made, pioglitazone (most efficacy data, but off label outside T2DM) or vitamin E (better safety and tolerability in the short term) or their combination could be used for NASH*

A total of 66% of EU5 physicians, 90% of Middle Eastern physicians, and 38% of Canadian physicians said they were likely to initiate pharmacological treatment. Overall, 1,510 patients (67%) currently received off-label treatment for NASH or associated underlying conditions with a specific stated aim of an intention to improve NASH (EU5, n = 1,206 [66%]; Canada, n = 49 [38%]; Middle East, n = 255 [87%]; *p* <0.0001). The most commonly prescribed drugs were statins, metformin, and vitamin E (Table S3). Physicians were not asked who had instituted these treatments and it was not clear whether motivation was based on a desire to improve general metabolic status or in the expectation of a specific liver-directed benefit. EU5 physicians more frequently targeted NASH and/or associated comorbidities using antidiabetic agents associated with weight loss, *e.g.* glucagon-like peptide 1 agonists, whereas Middle Eastern physicians more commonly used statins, pioglitazone, and vitamin E.

Among 1,263 patients with T2DM, the most common antidiabetic medications prescribed for managing NASH were metformin, incretin mimetics/glucagon-like peptide 1 agonists, and sodium glucose co-transporter-2 inhibitors; pioglitazone was only used by 8% of patients (Table S4).

Of 507 patients (22%) taking vitamin E, 37 (7%) had cirrhosis and 262 (52%) had a diagnosis of T2DM and might be considered unsuitable for such treatment as these groups were excluded from the PIVENS study.^24^

Non-pharmacological therapies were not widely used by patients, as reported by physicians. Overall, physicians reported that 147 patients (6%) used dietary supplements, 127 (6%) used herbal remedies, 25 patients (1%) used acupuncture, and 12 (0.5%) used hypnosis.

## Discussion

This real-world study of physicians managing patients with NASH in the EU5, Canada, and the Middle East highlights substantial variations in practice between regions. Data reveal substantial disconnects between recommendations in the cross-specialty EASL-EASD-EASO guidelines,^18^ which might be considered a reasonable standard of care, and the diagnosis and management of patients with NAFLD/NASH in routine clinical practice. These guidelines are broadly aligned with the guidelines developed by the Asian Pacific Association for the Study of the Liver^25^ and, with the exception of statement #1 (screening in high-risk populations), AASLD guidelines.^12^ Therefore, these findings are of concern as it is well established that greater NASH-associated fibrosis correlates with worse all-cause and liver-related patient outcomes.^3^

Despite being recommended in routine work-up for patients with NASH, screening using liver enzymes was not universally undertaken. Likewise, ultrasound was suboptimally used, although this varied across regions. The use of a ‘liver screen’ to exclude alternative diagnoses is fundamental in assessing patients with suspected liver disease. In this study, the use of such tests was variably adopted, with exclusionary tests for alternative or co-existent etiologies being inconsistently implemented. Crucially, guideline recommendations to screen for and treat important cardiovascular risk factors, *e.g.* T2DM and dyslipidemia, were not well adhered to. Cardiovascular disease is the leading cause of death in patients with NAFLD,^26^ yet only three-quarters of patients in our study cohort had undergone total cholesterol testing.

NAFLD affects up to 15% of the adult general population.^27^ In the face of such a large at-risk population, NITs can be used to risk-stratify patients and particularly to identify patients at low risk of advanced fibrosis and cirrhosis. Awareness and use of the simple, low-cost, and non-invasive FIB-4 test was minimal as reported by physicians, despite high rates of testing for individual components of the score (ALT, AST, and platelet count). Further education regarding its utility and ease of implementation may increase the use of this test, which has good positive and negative predictive values in patients with NAFLD and may act as a simple and efficient pre-screening tool, avoiding unnecessary liver biopsies^28^ and informing referral-to-specialist pathways when appropriate. Others have reported that NITs can reduce the need for liver biopsy to discriminate advanced fibrosis caused by NASH^15^^,^^29^ and that NITs in primary care could reduce unnecessary referrals to specialist liver centers.^30^ Close collaboration between specialties, including general practitioners and diabetologists, will be needed to maximize NIT use in routine clinical practice to enable timely identification of asymptomatic patients with occult advanced liver disease and facilitate referral to liver specialists.^31^

Guidelines recommend lifestyle interventions to induce weight loss through dietary modification and physical activity/exercise in managing NASH.^12^^,^^18^ This was commonly advocated by physicians across regions, although it is unknown what interventions were used nor whether this was simply general advice or through a multifaceted approach to support long-term behavior change. Targeted support has been deemed integral to patients with NASH successfully achieving and maintaining lifestyle changes.^7^^,^^32^ Almost half of all patients in our study were not referred to a professional nutritionist/dietician/health coach and may not have received enough support to make necessary lifestyle changes.

Pharmacological treatment recommendations for patients with NASH are currently limited, although efficacy data are available to support the use of pioglitazone and vitamin E in NASH.^24^ There are, however, no data to support the use of either approach in established cirrhosis. Furthermore, a proportion of patients in this study taking vitamin E had T2DM and so might be considered unsuitable for this treatment as such patients were excluded from the PIVENS study.^24^ Despite the absence of any regulatory-approved pharmacological treatments, Middle Eastern physicians were most likely to attempt pharmacological approaches targeting underlying disease causes. The motivation for this cannot be addressed through our dataset but may be related to patients requesting pharmacological intervention in the hope of rapidly improving their condition.

Treatment with statins was common in all regions, but less than might have been expected given the level of comorbidity in these patients. Indeed, the proportion of patients deemed at highest risk of cardiovascular events not receiving statin therapy was substantially greater than might be anticipated based on expected rates of statin intolerance.^33^ These findings suggest that, although evidence demonstrates that NASH increases patient cardiometabolic risk and that statins are safe in NAFLD/NASH,^34^^,^^35^ some prescribers remain reluctant to institute this potentially life-saving treatment. Contrary to guidelines,^18^ which reinforce the message that statins should be used, they are often discontinued by primary care physicians and diabetologists because of toxicity concerns.^36^^,^^37^ This may unwittingly be directly contributing to the risk of cardiovascular-related events. Our findings highlight the need for further education of physicians on this point.

The use of liver biopsy is recommended to aid the diagnosis of NASH in selected cases after risk stratification using NITs.^12^^,^^18^ Overall, liver biopsies were performed in fewer than 50% of patients with use greatest in the EU5. Lower liver biopsy rates could indicate effective screening practices preventing unnecessary biopsies or inclusion of a high proportion of patients who had either completed treatment in or failed to be included in a clinical trial. The large proportions of low-risk patients having a biopsy and high-risk patients who had no biopsy suggest that pre-screening NITs were not being used to guide decision-making for biopsy or NITs were being incorrectly interpreted.

Some limitations of this analysis warrant consideration. Participating physicians were more likely to be aware of NASH and have an interest in its management than the general physician population in secondary care. Inclusion of diabetologists was limited, with none in Canada and few in the Middle East being recruited. Further research is needed to more clearly differentiate management approaches according to specialist type. Currently, management options may be dictated by differences in healthcare systems, specifically availability of services locally (*e.g.* access to dieticians or exercise specialists) or secondary care protocols (*e.g.* governing patient eligibility for newer agents in T2DM), as well as reimbursement criteria. Identification of patients with NASH was based on physician judgment and not a formalized diagnostic checklist; this is representative of physicians’ real-world classification of patients.

The aim of this analysis was to assess real-world clinical practice and compare this retrospectively to a standard of care represented by reference guidelines, regardless of whether clinicians were currently referring to guidelines or not. The well-documented lack of availability or consensus on care pathways[5], [19], [20], [38] made it difficult to conduct a universal assessment of the optimal standard of care for patients with NASH. Lack of availability coupled with extensive variation in national, and nationally endorsed, clinical practice guidelines for NAFLD^38^ drove a decision to use the cross-specialty EASL-EASD-EASO guidelines, which we considered to be amongst the most comprehensive guidelines currently available and specifically relevant to the majority of territories sampled. In addition, some aspects of the guidelines used in this analysis are optional; consequently, their implementation is not always clear-cut and some degree of ambiguity and personal choice in how they are interpreted and applied may result. Health system preparedness for the multidisciplinary management of patients with NASH is poor.^39^ National and international management guidelines and care pathways are clearly needed to optimize the diagnosis and management of patients with NASH.

In conclusion, real-world NASH management approaches differ substantially from those advocated in established reference guidelines. Moreover, management practices vary substantially between and within the EU5, Canada, and the Middle East. Regional differences in NASH management may be influenced by healthcare systems and cultural differences; these need to be accounted for across geographies. Low levels of observed adherence in real-world practice to multi-society professional guidelines advocating a standard of care clearly highlight a need for greater development of regional and national strategies, and training in assessment and management of NAFLD/NASH across all specialist groups and territories if patients are to receive optimum care.

## Financial support

This analysis was funded by 10.13039/100005564Gilead Sciences, Inc. Data collection was undertaken by Adelphi Real World as part of an independent survey, entitled the Adelphi NASH DSP. Gilead Sciences, Inc. did not influence the original survey through either contribution to the design of questionnaires or data collection. The analysis described here used data from the Adelphi NASH DSP. The DSP is a wholly owned Adelphi product. Gilead Sciences, Inc. are one of multiple subscribers to the DSP. QMA is supported by the 10.13039/100014461Newcastle NIHR Biomedical Research Centre and the LITMUS (Liver Investigation: Testing Marker Utility in Steatohepatitis) consortium funded by the Innovative Medicines Initiative (IMI2) Program of the 10.13039/100010661European Union under Grant Agreement 777377; this Joint Undertaking receives support from the 10.13039/100010661European Union’s Horizon 2020 research and innovation programme and 10.13039/100013322EFPIA.

## Authors’ contributions

QMA responsible for clinical oversight and guidance as lead author. Study set-up and data collection were led by VH. Analysis design was conducted by all authors with statistical analyses prepared by GM. JP and VH wrote the manuscript supported by a medical writer. All authors participated in manuscript development and finalization, assume responsibility for the accuracy and completeness of the data, and vouch for the study’s fidelity to the protocol.

## Data availability statement

All data relevant to the analysis are included in the article. All data that support the findings of this survey are the intellectual property of Adelphi Real World. Data are available upon reasonable request to Victoria Higgins at Victoria.Higgins@adelphigroup.com.

## Role of the funding source

Gilead Sciences, Inc. subscribed to the Adelphi NASH DSP and were involved in the analysis, design, and editing of the manuscript. Survey design, data collection, data analysis, statistical analyses, data interpretation, and editing were performed by Adelphi Real World. All authors had access to the aggregated data, provided critical feedback, and approved the final manuscript.

## Conflict of interest

Quentin M. Anstee is coordinator of the IMI2 LITMUS consortium, which is funded by the EU Horizon 2020 programme and EFPIA. This multi-stakeholder consortium includes industry partners. He reports research grant funding from Allergan/Tobira, AstraZeneca, GlaxoSmithKline, Glympse Bio, Novartis Pharma AG, Pfizer Ltd., Vertex; royalties/licenses from Elsevier; consultancy on behalf of Newcastle University for 89Bio, Allergan/Tobira, Altimmune, AstraZeneca, Axcella, Blade, BMS, BNN Cardio, Cirius, CymaBay, EcoR1, E3Bio, Eli Lilly & Company, Galmed, Genentech, Genfit, Gilead, Grunthal, HistoIndex, Indalo, Intercept, Inventiva, IQVIA, Janssen, Madrigal, MedImmune, Medpace, Metacrine, NGMBio, North Sea Therapeutics, Novartis, Novo Nordisk A/S, PathAI, Pfizer Ltd., Poxel, ProSciento, Raptor Pharma, Roche, Servier, Terns, The Medicines Company, Viking Therapeutics; and speaker fees from Abbott Laboratories, Allergan/Tobira, BMS, Clinical Care Options, Falk, Fishawack, Genfit SA, Gilead, Integritas Communications, Kenes, MedScape. Kate Hallsworth is supported by a National Institute for Health Research/Health Education England Clinical Lectureship (CAT CL-2013-04-010). James Piercy and Victoria Higgins are employees of Adelphi Real World. Niall Lynch, Adrien Hauvespre, Eid Mansour, and Sam Kozma are employees of Gilead. John-Paul Marino was employed by and owned stock in Gilead at the time of the research. Juliana Bottomley has received consultancy payments from Gilead.

Please refer to the accompanying ICMJE disclosure forms for further details.
