# Supplementary material for: Real-world management of non-alcoholic steatohepatitis differs from clinical practice guideline recommendations and across regions
Source: JHEP Rep. 2021 Nov 22;4(1):100411. doi: 10.1016/j.jhepr.2021.100411 (PMC8686034; doi:10.1016/j.jhepr.2021.100411)
Supplement: Multimedia component 1 [file mmc1.pdf]

Real-world management of non-alcoholic steatohepatitis differs from clinical practice guideline recommendations and across regions

Quentin M. Anstee, Kate Hallsworth, Niall Lynch, Adrien Hauvespre, Eid Mansour, Sam Kozma, John-Paul Marino, Juliana Bottomley, James Piercy, Victoria Higgins

Table of contents

Supplementary results... 2

Table S1... 4

Table S2... 6

Table S3... 9

Table S4... 10

Supplementary results

Patients and physicians

Diabetologists provided the fewest patients in all regions; Canadian endocrinologists did not declare management of enough patients with non-alcoholic steatohepatitis (NASH) to qualify for the study; only two Middle East diabetologists (of 41 specialists) provided patient data. Notably, although hepatologists and gastroenterologists managed some patients with NASH presenting with type 2 diabetes mellitus (Middle East, 47% and 40%, respectively; EU5 [France, Germany, Italy, Spain, and United Kingdom], 51% and 52%, respectively), diabetologists were more likely to manage these patients (Middle East, 75%; EU5, 78%).

Diagnosis and monitoring

Physicians used a variety of investigations to help work-up, diagnose, and/or stage NASH. Visual examination of patients identified a range of symptoms commonly observed in patients with NASH and more common in those with advanced NASH (Supplementary Table S1). Tests performed included diagnostic tests and those designed to eliminate the contribution of patient lifestyle, fatty liver diseases, autoimmune conditions, and other metabolic conditions. Testing patterns varied across the regions and according to fibrosis score (Supplementary Table S2). Tests more commonly performed in patients with advanced fibrosis included vibration-controlled transient elastography and liver biopsy, the latter having more pronounced usage among patients with advanced fibrosis in the Middle East. Patients with advanced fibrosis underwent more tests than those with early fibrosis (Supplementary Table S2).

Composite scores to stratify stage of fibrosis were most likely to be employed by clinicians in the EU5 (641 of 1411 patients [45%] assigned a composite score; Supplementary Table S2) and least likely in Canada (21 of 90 patients; 23% of patients).

Table S1. Symptoms according to current physician-reported fibrosis score.

	EU5 (n=1468)			Canada (n=130)			Middle East (n=293)		
Symptom, n (%)	F0–2 (n=1147)	F3/4 (n=321)	p-value	F0–2 (n=47)	F3/4 (n=35)	p-value	F0–2 (n=195)	F3/4 (n=68)	p-value
Mean no. of current symptoms (SD)	**2.8 (2.5)**	**3.3 (2.7)**	0.0017[a]	**1.3 (1.3)**	**2.7 (2.5)**	0.0016[a]	**2.1 (1.3)**	**2.7 (1.2)**	0.0004[a]
Symptoms, n (%)									
Fatigue	653 (57)	192 (60)	0.3715[b]	**23 (49)**	**27 (77)**	0.0122[b]	**148 (76)**	**66 (97)**	<0.0001[b]
Sleep disturbance	422 (37)	131 (41)	0.1930[b]	9 (19)	13 (37)	0.0822[b]	**17 (9)**	**9 (13)**	0.3444[b]
General weakness	387 (34)	127 (40)	0.0552[b]	13 (28)	14 (40)	0.3422[b]	**128 (66)**	**61 (90)**	0.0001[b]
Unexplained weight loss	34 (3)	15 (5)	0.1578[b]	0	1 (3)	0.4268[b]	4 (2)	1 (1)	1.0000[b]
Aching/discomfort in upper right abdomen	314 (27)	69 (21)	0.0370[b]	7 (15)	5 (14)	1.0000[b]	29 (15)	10 (15)	1.0000[b]
Jaundice	**51 (4)**	**25 (8)**	0.0221[b]	0	1 (3)	0.4268[b]	2 (1)	1 (1)	1.0000[b]
Itchy skin	183 (16)	59 (18)	0.3076[b]	0	1 (3)	0.4268[b]	7 (4)	0	0.1957[b]
Swelling in the legs, ankles, feet	274 (24)	94 (29)	0.0580[b]	**1 (2)**	**8 (23)**	0.0040[b]	**20 (10)**	**14 (21)**	0.0361[b]
Swelling of the stomach/abdomen	259 (23)	82 (26)	0.2632[b]	**1 (2)**	**6 (17)**	0.0384[b]	20 (10)	5 (7)	0.6329[b]
Tendency to bleed and bruise more easily	79 (7)	22 (7)	1.0000[b]	**0**	**4 (11)**	0.0299[b]	0	0	
Hair loss	84 (7)	26 (8)	0.6323[b]	1 (2)	1 (3)	1.0000[b]	5 (3)	1 (1)	1.0000[b]
Fever and shivering attacks	**40 (3)**	**27 (8)**	0.0004[b]	0	1 (3)	0.4268[b]	2 (1)	0	1.0000[b]
Insomnia	**161 (14)**	**66 (21)**	0.0052[b]	5 (11)	4 (11)	1.0000[b]	7 (4)	7 (10)	0.0543[b]

Changes to personality	50 (4)	21 (7)	0.1076[b]	0	2 (6)	0.1792[b]	0	1 (1)	0.2586[b]
Memory loss	**48 (4)**	**32 (10)**	0.0001[b]	0	2 (6)	0.1792[b]	1 (1)	1 (1)	0.4510[b]
Confusion/difficulty concentrating	**114 (10)**	**51 (16)**	0.0037[b]	1 (2)	4 (11)	0.1583[b]	17 (9)	8 (12)	0.4752[b]
NASH condition status, n (%)	*(n=1147)*	*(n=321)*	<0.0001[c]	*(n=47)*	*(n=35)*	<0.0001[c]	*(n=195)*	*(n=68)*	<0.0001[c]
Improving	**202 (18)**	**19 (6)**		**14 (30)**	**1 (3)**		**95 (49)**	**16 (24)**	
Stable	**730 (64)**	**150 (47)**		**31 (66)**	**17 (49)**		**84 (43)**	**16 (24)**	
Deteriorating slowly	**188 (16)**	**119 (37)**		**2 (4)**	**12 (34)**		**13 (7)**	**33 (49)**	
Deteriorating rapidly	**10 (1)**	**23 (7)**		**0**	**4 (11)**		**0**	**1 (1)**	
Fluctuating	**17 (1)**	**10 (3)**		**0**	**1 (3)**		**3 (2)**	**2 (3)**	

NOTE: Values in bold indicate statistically significant differences between the F0–2 and F3/4 groups. [a]T-test. [b]Fishers exact test. [c] Chi^2 test.

EU5, France, Germany, Italy, Spain, and UK; NASH, non-alcoholic steatohepatitis; SD, standard deviation.

Table S2. Diagnostic and monitoring tests used in NASH according to geographical region and patient's fibrosis score at diagnosis.

	EU5 (n=1411)		Canada (n=90)		Middle East (n=272)	
**Physician-stated fibrosis score**	**F0-F2**	**F3/F4**	**F0-F2**	**F3/F4**	**F0-F2**	**F3/F4**
**Diagnostic tests**	*(n=1079)*	*(n=332)*	*(n=49)*	*(n=41)*	*(n=222)*	*(n=50)*
**Elimination tests/assessments**	**982 (91)**	**315 (95)**	**47 (96)**	**41 (100)**	**208 (94)**	**50 (100)**
High BMI range: >30 kg/m^2	684 (63)	203 (61)	37 (76)	33 (80)	187 (84)	45 (90)
Alcohol intake (e.g. <20 g/day in women and <30 g/day in men)	740 (69)	244 (73)	44 (90)	38 (93)	21 (9)	3 (6)
Hypertension (BP >140/90 mmHg)	610 (57)	201 (61)	28 (57)	34 (83)	185 (83)	47 (94)
Gilbert's syndrome	310 (29)	81 (24)	21 (43)	19 (46)	27 (12)	2 (4)
Paget's disease	202 (19)	46 (14)	7 (14)	7 (17)	19 (9)	1 (2)
Wilson's disease	357 (33)	96 (29)	27 (55)	26 (63)	42 (19)	4 (8)
Celiac disease	376 (35)	80 (24)	24 (49)	19 (46)	32 (14)	1 (2)
Serum ferritin/transferrin saturation	671 (62)	221 (67)	41 (84)	35 (85)	23 (10)	5 (10)
Alpha-1 antitrypsin	455 (42)	130 (39)	41 (84)	34 (83)	9 (4)	0
Serum ceruloplasmin	409 (38)	132 (40)	38 (78)	29 (71)	53 (24)	12 (24)
Impaired fasting glucose	497 (46)	162 (49)	30 (61)	21 (51)	89 (40)	18 (36)
HbA_{1c}	761 (71)	239 (72)	39 (80)	32 (78)	133 (60)	35 (70)
Presence of type 2 diabetes	671 (62)	215 (65)	32 (65)	26 (63)	167 (75)	45 (90)
Presence of type 1 diabetes	0	0	0	0	0	0
Raised fasting triglycerides	741 (69)	199 (60)	39 (80)	27 (66)	183 (82)	50 (100)
HDL-cholesterol	802 (74)	229 (69)	37 (76)	30 (73)	200 (90)	50 (100)
LDL-cholesterol	790 (73)	221 (67)	37 (76)	31 (76)	199 (90)	50 (100)
Total cholesterol	820 (76)	233 (70)	36 (73)	29 (71)	198 (89)	48 (96)
**Liver function blood tests**	**919 (85)**	**254 (77)**	**48 (98)**	**40 (98)**	**198 (89)**	**50 (100)**
Aspartate aminotransferase	809 (75)	220 (66)	45 (92)	35 (85)	197 (89)	50 (100)
Alanine aminotransferase	854 (79)	236 (71)	48 (98)	38 (93)	197 (89)	50 (100)
AST:ALT ratio	217 (20)	62 (19)	12 (24)	13 (32)	192 (86)	49 (98)

Alkaline phosphatase	564 (52)	161 (48)	42 (86)	35 (85)	196 (88)	49 (98)
Gamma-glutamyltransferase	670 (62)	172 (52)	30 (61)	21 (51)	195 (88)	49 (98)
Platelets	581 (54)	178 (54)	43 (88)	36 (88)	195 (88)	49 (98)
INR ratio	343 (32)	103 (31)	37 (76)	30 (73)	195 (88)	49 (98)
Serum albumin	510 (47)	145 (44)	38 (78)	33 (80)	193 (87)	50 (100)
Total bilirubin	537 (50)	174 (52)	42 (86)	37 (90)	166 (75)	48 (96)
**Imaging tests**	**980 (91)**	**294 (89)**	**36 (73)**	**31 (76)**	**210 (95)**	**49 (98)**
Ultrasonography (e.g. CEUS, ARFI)	904 (84)	264 (80)	35 (71)	28 (68)	210 (95)	49 (98)
Computed tomography	152 (14)	89 (27)	4 (8)	6 (15)	83 (37)	38 (76)
Magnetic resonance imaging	234 (22)	68 (20)	2 (4)	3 (7)	2 (1)	2 (4)
Proton magnetic resonance spectroscopy	17 (2)	2 (1)	0	0	1 (<1)	0
**Other tests**						
Liver biopsy	513 (48)	184 (55)	25 (51)	15 (37)	23 (10)	34 (68)
VCTE™	670 (62)	230 (69)	27 (55)	24 (59)	162 (73)	40 (80)
**Composite scores**	**487 (45)**	**154 (46)**	**13 (27)**	**8 (20)**	**80 (36)**	**17 (34)**
NAFLD Activity Score	200 (19)	72 (22)	3 (6)	5 (12)	2 (1)	0
Steatosis Activity Fibrosis Score	61 (6)	24 (7)	0	0	10 (5)	3 (6)
SteatoTest	53 (5)	22 (7)	0	0	8 (4)	7 (14)
ActiTest	47 (4)	29 (9)	0	0	26 (12)	8 (16)
FibroTest	109 (10)	55 (17)	2 (4)	2 (5)	67 (30)	10 (20)
Serum Enhanced Liver Fibrosis test	11 (1)	7 (2)	0	1 (2)	3 (1)	0
NAFLD Fibrosis Score	95 (9)	35 (11)	8 (16)	1 (2)	9 (4)	2 (4)
NAFLD Liver Fat Score	20 (2)	6 (2)	0	0	2 (1)	3 (6)
Fibrosis-4 Index	61 (6)	24 (7)	3 (6)	2 (5)	2 (1)	4 (8)
BARD score	17 (2)	5 (2)	2 (4)	2 (5)	3 (1)	6 (12)
Fatty Liver Index	47 (4)	19 (6)	0	0	2 (1)	1 (2)
Serological tests showing absence of viral hepatitis	765 (71)	266 (80)	41 (84)	36 (88)	180 (81)	48 (96)
Antimitochondrial antibody	632 (59)	200 (60)	40 (82)	35 (85)	94 (42)	10 (20)
Antinuclear antibody	638 (59)	201 (61)	42 (86)	38 (93)	111 (50)	19 (38)
Liver kidney microsomal type 1 antibody	573 (53)	207 (62)	26 (53)	19 (46)	107 (48)	28 (56)
Anti-neutrophil cytoplasmic antibodies	486 (45)	141 (42)	27 (55)	14 (34)	122 (55)	28 (56)
Tissue transglutaminase IgA and/or IgG	478 (44)	140 (42)	27 (55)	23 (56)	98 (44)	31 (62)
**Monitoring tests**	*(n=1147)*	*(n=321)*	*(n=47)*	*(n=35)*	*(n=195)*	*(n=68)*
Mean no. of monitoring tests (SD)	5.7 (3.5)	6.3 (3.6)	5.6 (2.9)	6.3 (3.4)	9.6 (4.0)	12.0 (2.0)

NOTE: Values in bold indicate statistically significant differences between the F0–2 and F3/4 groups.

ALT, alanine aminotransferase; ARFI, acoustic radiation force impulse; AST, aspartate aminotransferase; BARD, BMI, AST:ALT ratio, and diabetes; BMI, body mass index; BP, blood pressure; CEUS, contrast-enhanced ultrasound; EU5, France, Germany, Italy, Spain, and UK; HbA_{1c}, glycated hemoglobin; HDL, high-density lipoprotein; Ig, immunoglobulin; INR, international normalized ratio; LDL, low-density lipoprotein; NAFLD, non-alcoholic fatty liver disease; VCTE, vibration-controlled transient elastography.

Table S3. Medications used specifically for NASH and/or to treat associated conditions in an attempt to control the patient's NASH.

Medication, n (%)	Overall (n=2267)	EU5 (n=1844)	Canada (n=130)	Middle East (n=293)	*p* value
Treatment status					
On treatment[a]	1510 (67)	1206 (65)	49 (38)	255 (87)	<0.0001
Not on treatment	729 (32)	611 (33)	80 (62)	38 (13)	<0.0001
Don't know	28 (1)	27 (1)	1 (1)	0	<0.0001
Treatments					
Statins	854 (38)	662 (36)	20 (15)	172 (59)	<0.0001
Metformin	793 (35)	667 (36)	21 (16)	105 (36)	<0.0001
Vitamin E	507 (22)	346 (19)	16 (12)	145 (49)	<0.0001
GLP-1	268 (12)	262 (14)	4 (3)	2 (1)	<0.0001
DPP-4	185 (8)	116 (6)	2 (2)	67 (23)	<0.0001
SGLT-2	148 (7)	137 (7)	1 (1)	10 (3)	0.0008
Pioglitazone	142 (6)	89 (5)	4 (3)	49 (17)	<0.0001
Vitamin C	98 (4)	94 (5)	2 (2)	2 (1)	0.0007
Orlistat	77 (3)	57 (3)	0	20 (7)	0.0004
PCSK9	12 (1)	12 (1)	0	0	0.2507
Other	166 (7)	65 (4)	2 (2)	99 (34)	<0.0001

DPP-4, dipeptidyl peptidase-4; EU5, France, Germany, Italy, Spain, and UK; GLP-1, glucagon-like peptide 1; PCSK9, proprotein convertase subtilisin/kexin type 9; SGLT-2, sodium glucose co-transporter-2.

[a]Treatment was defined as currently taking vitamin C, vitamin E, pioglitazone, orlistat, GLP-1, DPP-4, SGLT-2, statins, PCSK9, metformin, other medication aiming either to treat NASH off label and/or to treat associated conditions in an attempt to control NASH.

Table S4. Pharmacological treatment of patients with NASH and type 2 diabetes.

Medication, n (%)	Overall (n=1263)	EU5 (n=1083)	Canada (n=51)	Middle East (n=129)	*p* value
Metformin	664 (53)	571 (53)	18 (35)	75 (58)	0.0211
Statins	582 (46)	473 (44)	10 (20)	99 (77)	<0.0001
GLP-1	252 (20)	246 (23)	4 (8)	2 (2)	<0.0001
Vitamin E	262 (21)	198 (18)	5 (10)	59 (46)	<0.0001
SGLT-2	140 (11)	132 (12)	1 (2)	7 (5)	0.0073
DPP-4	171 (14)	107 (10)	2 (4)	62 (48)	<0.0001
Pioglitazone	102 (8)	68 (6)	3 (6)	31 (24)	<0.0001
Orlistat	55 (4)	46 (4)	0	9 (7)	0.1064
Other	74 (6)	35 (3)	1 (2)	38 (29)	<0.0001

DPP-4, dipeptidyl peptidase-4; EU5, France, Germany, Italy, Spain, and UK; GLP-1, glucagon-like peptide 1; SGLT-2, sodium glucose co-transporter-2.
